# Diverse Effects of Lysophosphatidic Acid Receptors on Ovarian Cancer Signaling Pathways

**DOI:** 10.1155/2019/7547469

**Published:** 2019-09-17

**Authors:** Hadil Onallah, Ben Davidson, Reuven Reich

**Affiliations:** ^1^Institute of Drug Research, School of Pharmacy, Faculty of Medicine, The Hebrew University of Jerusalem, Jerusalem 91120, Israel; ^2^Department of Pathology, Oslo University Hospital, Norwegian Radium Hospital, Oslo, Norway; ^3^Faculty of Medicine, Institute of Clinical Medicine, University of Oslo, Oslo, Norway

## Abstract

Lysophosphatidic acid (LPA) is a bioactive phospholipid with mitogenic and growth factor-like activities affecting cell invasion, cancer progression, and resistance. It is produced mainly by autotaxin and acts on six G-protein-coupled receptors, LPAR1-6. LPA has recently been implicated as a growth factor present in ascites of ovarian cancer patients. However, mitogenic pathways stimulated by LPA via its receptors may involve any novel, thus far uncharacterized, signaling pathway(s). Here we show that three LPA receptors are involved in tumor progression by activation of both the AKT and ERK signaling pathways. CRISPR-edited LPAR2 and LPAR3 knockouts have opposing effects on ERK activation, whereas LPAR6 is involved in the activation of AKT, affecting cell migration and invasion. Our study identifies specific molecular machinery triggered by LPA and its receptors that modulates tumor cells and can serve as therapeutic target in this malignancy.

## 1. Introduction

Lysophosphatidic acid (LPA) is a phospholipid produced by autotaxin (ATX) that activates six G-protein-coupled receptors (LPAR1-6) [1–3]. High levels of LPA are detected in the plasma and ascites fluid of ovarian cancer (OC) patients, and LPA is thus regarded as a novel ovarian cancer-activating factor [4–7]. OC, particularly the most common histotype, high-grade serous carcinoma (HGSC), is characterized by diagnosis at an advanced stage, often with the formation of malignant effusions within the peritoneal and pleural cavities, resulting in a five-year survival rate of 45% for all cases [8–10]. The relationship between LPA and OC is well established, and previous reports have suggested that upregulated expression of LPARs may be involved in the mechanism underlying tumor growth and metastasis in this cancer [11–20]. Yet little is known about the specific role of each one of these receptors and its downstream effect. Elucidating the signaling pathway of each LPAR may help in further understanding the ATX-LPA axis and its involvement in OC progression.

In a recent paper, we reported that LPAR2, LPAR3, and LPAR6 are differentially expressed at different anatomic sites in HGSC and identified a prognostic role for LPAR1, LPAR2, and LPAR5 levels in effusion specimens [21]. Here, we genetically engineered OC knockout (KO) cells for three of these receptors and studied their role in a 3D cell line model mimicking malignant effusions. Our results indicate that while LPAR3 stimulates the activation of ERK, LPAR2 inhibits its activity and LPAR6 stimulates the activation of the AKT pathway. All three receptors promote OC invasion, whereas LPAR3 and LPAR6 promote cell migration. Taken together with the finding that the ATX-LPA axis is involved in OC progression and related to disease outcome, our data suggest an important role for LPARs in this malignancy.

## 2. Materials and Methods

### 2.1. Cell Lines

The OVCAR3 and ES-2 OC cell lines were obtained from the American Type Culture Collection (ATCC; Manassas, VA) and cultured in the appropriate media according to the manufacturer's instructions (obtained from Biological Industries, Beit-Haemek, Israel). The medium was supplemented with 1% L-glutamine, 1% sodium pyruvate, 1% vitamin solution, 1% nonessential amino acids, and 10% fetal calf serum. All cells were grown in a humidified atmosphere of 95% air and 5% CO_2_.

### 2.2. 3D Spheroid Cell Line Model

**Figure 1:**
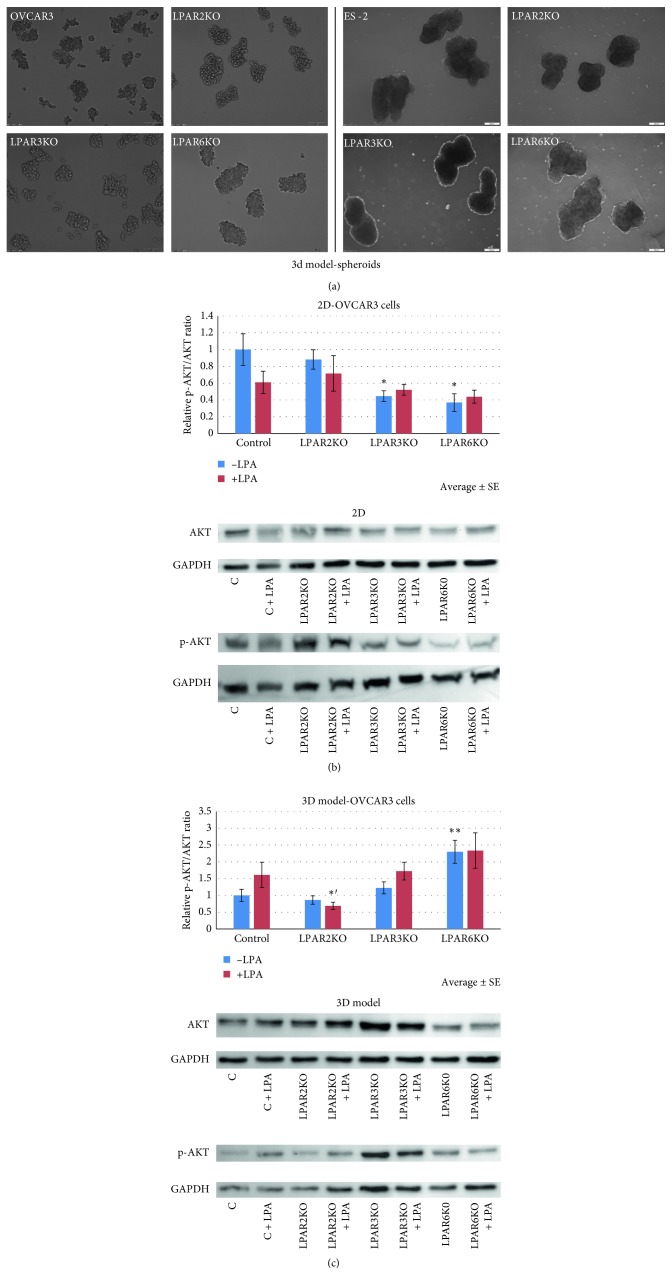
AKT and ERK expression is altered in LPAR OVCAR3 KO cells. (a) A representative photo of OVCAR3 and ES2 spheroids. (b)-(c) The p-AKT/AKT ratio is elevated in LPAR6KO cells in the 3D model (*p* < 0.01) and decreased in the 2D model compared with control cells (*p* < 0.05). In addition, LPAR3KO cells cultured in 2D show decreased p-AKT levels (*p* < 0.05). Average ± SE; ^*∗*^*p* < 0.05, ^*∗∗*^*p* < 0.01, and ^*∗*′^*p* < 0.05 compared with treated control (Student's *t*-test). Group size: 3D untreated: control *n* = 14, LPAR2KO *n* = 13, LPAR3KO *n* = 14, LPAR6KO *n* = 16; treated: control *n* = 8, LPAR2KO *n* = 7, LPAR3KO *n* = 8, LPAR6KO *n* = 9; 2D untreated: control *n* = 3, LPAR2KO *n* = 5, LPAR3KO *n* = 4, LPAR6KO *n* = 4; treated: control *n* = 4, LPAR2KO *n* = 4, LPAR3KO *n* = 5, LPAR6KO *n* = 5.

To generate spheroids, OVCAR3 and ES-2 cells were dissociated by trypsinization and suspended, following which 4 × 10^5^ cells/well were placed for agitation in a 6-well plate in order to prevent cell attachment to the culture plate and to facilitate spheroid formation (Figure 1(a)). Cells were treated with 10 *μ*M LPA (#10010093, Cayman Chemicals) 24 hours later, and spheroids were collected for experiments 24 hours after adding LPA (total of 48 hours of agitation).

### 2.3. Western Blotting (WB)

Twenty-five micrograms of protein were resolved by 10% SDS-PAGE. The separated extracts were transferred onto Immobilon PVDF membrane (Millipore, Bedford, MA) in a transfer buffer. Membranes were then incubated for 1 h in TBST containing 5% Difco™ skim milk (BD Biosciences, San Jose, CA) to block nonspecific binding. Blots were then incubated with ERK monoclonal antibody (#4695, Cell Signaling Biotechnology, Danvers, MA), p-ERK monoclonal antibody (#4377, Cell Signaling Biotechnology), AKT monoclonal antibody (#4691, Cell Signaling Biotechnology), and p-AKT monoclonal antibody (#4060, Cell Signaling Biotechnology). GAPDH (14C10; Cell Signaling Biotechnology) was used as loading control. Proteins were detected using EZ-ECL Chemiluminescence detection kit for HRP (Biological Industries) according to the manufacturer's specifications using Image Lab 5.0 gel reader (Bio-Rad, Hercules, CA).

Densitometer analysis of blots was performed using a computerized image analysis program (Image-J, NIH, Bethesda, MD). Protein expression levels were established by calculating the target molecule/GAPDH ratio (all cases scored for band intensity compared with internal control). Expression intensity of 5% or less of control levels was interpreted as negative.

### 2.4. Plasmid Construction

Restriction enzymes (BbsI) and T4 DNA ligase (Quick Ligation Kit) were purchased from New England Biolabs, Inc (Beverly, MA) and used with the recommended buffer. For extraction of PCR products from agarose gel or purification of PCR products, the Nucleospin® Gel and PCR Clean-up Kit (Macherey-Nagel, Berlin, Germany) was used.

A 25-nucleotide genomic target sequence was selected from Genome-Browser for each one of the three genes. The forward and reverse oligos were mixed to a final concentration of 50 *μ*M and annealed in a thermocycler according to the following program: 95°C for 5 minutes, followed by ramping to 25°C at 5°C/minute. The annealed mix of oligos was ligated into the CRISPR plasmid vector pSpCas9(BB)-2A-GFP(PX458) plasmid #48138. Vectors were generously provided by Prof. Yehudit Bergman at the Hebrew University of Jerusalem. pX458 was digested with BbsI prior to ligation. sgRNAs used in this method are listed in Table 1.

Plasmids were amplified in *DH5*-*α*-competent bacteria. The plasmids were mixed with 200 *μ*L of the bacteria, vortexed, incubated on ice for 20 minutes, heat shocked at 42°C for 45 seconds, and immediately placed on ice. The bacteria were then grown in LB media at 37°C, 200 rpm for 60 minutes, following which the cells were plated with appropriate selection. Plates were incubated at 37°C overnight. A few colonies were selected from the plates and were grown overnight at 37°C, 200 rpm in LB media with the appropriate selection. DNA was then extracted from the cultures using the Quick Plasmid Miniprep Kit (#K-3030; Invitrogen).

### 2.5. DNA Transfection

OVCAR3 and ES2 cells were seeded at a density of 5 × 10^5^ cells/well in 6-well culture plates. The following morning, the media was replaced and the foreign DNA (plasmid) was transfected into the cells by Lipofectamine transfection (Invitrogen, Carlsbad, CA) according to manufacturer instructions. Transfected cells were then sorted by flow cytometry for GFP (Aria II sorter, BD Biosciences).

### 2.6. Single-Cell Clonal Isolation

A cell suspension of OVCAR3 and ES2 cells transfected with the appropriate plasmid was prepared by serial dilution, with resulting concentration of 1 cell/100 *μ*L. 100 *μ*L of the cell suspension was added to wells preplated with 100 *μ*L of appropriate medium (in a 96-well plate). After two weeks, colonies were expanded into 48-well plates, then 24-well plates, 12-well plates, and finally 6-well plates, generating stable cell lines. Validation of knockout was done by WB, qRT-PCR, T7EI assay, and sequencing.

### 2.7. Wound Healing Assay

Cells were seeded to create a confluent monolayer 24 hours prior to the experiment. Monolayers were scraped with a pipette tip to create similar size scratches. Cells were incubated for 48 hours, supplemented with growth media with or without LPA, and images were acquired at 0, 24, and 48 hours. Scratch closure analysis was done by comparing images from the indicated times using an Axiocam 105 color microscope camera with magnification ×10 (Zeiss, Oberkochen, Germany).

### 2.8. Boyden Chamber Invasion Assay

Matrigel (reconstituted basement membrane; 25 *μ*g) was dried on a polycarbonated filter (PVP-free, Nuclepore, Whatman, Maidstone, UK). Fibroblast-conditioned medium (obtained from confluent NIH-3T3 cells cultured in serum-free DMEM) was used as chemoattractant. Cells were harvested by brief exposure to 1 mM EDTA, washed with DMEM containing 0.1% bovine serum albumin, with or without LPA, and added to the Boyden chamber (200,000 cells). To study the effect of ERK, the MEK inhibitor U0126 (V112A, Promega) was used at a 0.5 *μ*M concentration, according to the manufacturer's instructions. Cells that traversed the Matrigel layer and attached to the lower surface of the filter were stained with Diff-Quik kit (Dade Diagnostics, Aguada, PR) and counted in five random fields. The mean was calculated for each cell line and expressed as mean ± SE.

### 2.9. Statistical Analysis

Statistical analysis of treatment results with LPA and knockouts *in vitro* was performed using Student's *t*-test.

## 3. Results

### 3.1. LPAR-Knockout Cells Have Different AKT and ERK Activation Levels in OVCAR3 Cells

The functional role of three LPAR family members was investigated in OC cells *in vitro*. To provide a clean background for the expression of structure-based point mutants, we used clustered, regularly interspaced, short palindromic repeat (CRISPR) technology to knock out LPAR2, LPAR3, and LPAR6.

Several studies identified LPA as a modulator of ERK, a member of the MAPK family regulating cell migration and invasion [22, 23]. Furthermore, various works identified AKT as a key role player in LPA-induced cell migration [24], and its invasion pathway has been found to be upregulated in OC [8], making both AKT and ERK potentially relevant pathways for studying the effect of LPA on this malignancy.

Previous studies have demonstrated that OC patients have high LPA plasma levels, ranging between 0.1 *μ*M and 10 *μ*M, with a mean of 8.6 *μ*mol/L [1, 4]. Brusevold et al. used 10 *μ*M LPA and showed that LPA induced a strong dose-dependent migratory response with this concentration [25]. Several other studies have applied the same concentration to cell line experiments, including analysis of OVCAR3 cells, the same cell line used in the present study [26, 27]. Concentration of 10 *μ*M LPA was therefore chosen as the preferred dose for all experiments.

The signaling pathway of each LPAR was assessed in two cell culture models, 2D and spheroids (3D; Figure 1(a)), the latter mimicking tumor cell growth in effusions and ascites in OC, thus making this model a more accurate way to study the malignancy in its 3D form. While analyzing the OVCAR3 cell line in the 2D form, a decrease in p-AKT/AKT ratio was found for both LPAR3KO and LPAR6KO, with no effect by LPA stimulation. Cells cultured in 3D form expressed elevated levels of p-AKT in LPAR6KO cells. Furthermore, upon treatment with 10 *μ*M LPA, LPAR2KO cells expressed a decrease in p-AKT compared with control cells treated with LPA (Figures 1(b) and 1(c)). These results suggest that LPAR6 may be responsible for AKT activation.

### 3.2. LPAR2KO and LPAR3KO Cells Have opposite Effects on p-ERK in OVCAR3 Spheroids

**Figure 2:**
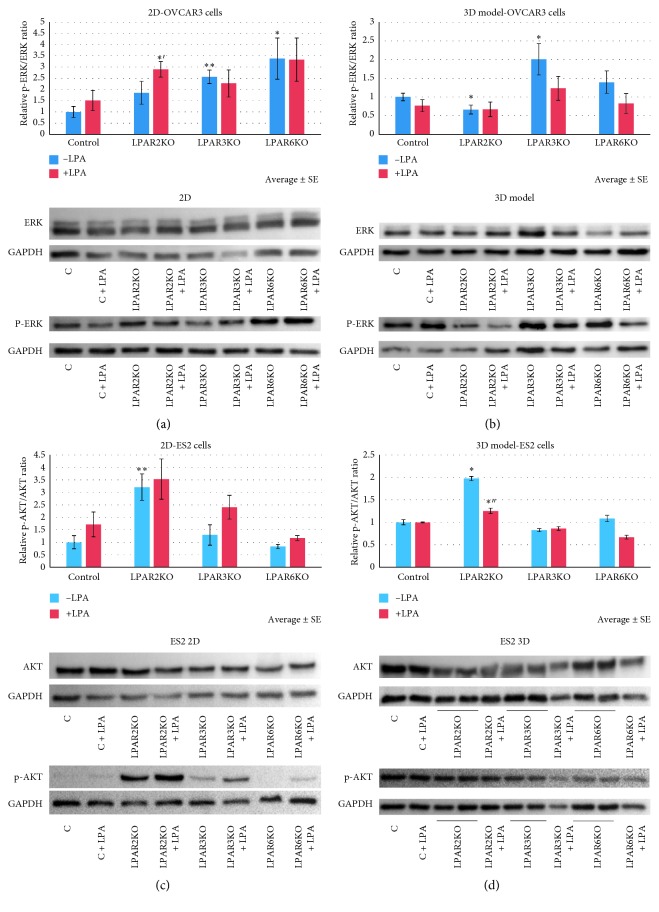
AKT and ERK expression is altered in LPAR OVCAR3 KO cells. (a) Protein levels of p-ERK/ERK in 2D OVCAR3 cell line. p-ERK/ERK ratio is elevated in LPAR3KO (*p* < 0.01) and LPAR6KO (*p* < 0.05) cells compared with untreated control cells and elevated in LPAR2KO upon LPA stimulation compared with treated controls (*p* < 0.05). (b) Protein levels of p-ERK/ERK in the 3D model of OVCAR3 cell line. p-ERK/ERK ratio is elevated in spheroids of LPAR3KO yet decreased in spheroids of LPAR2KO (*p* < 0.05) compared with untreated control cells. Average ± SE; ^*∗*^*p* < 0.05, ^*∗∗*^*p* < 0.01, and ^*∗*′^*p* < 0.05 compared with treated control (Student's *t*-test). Group size: 2D: control *n* = 4, LPAR2KO *n* = 5, LPAR3KO *n* = 4, LPAR6KO *n* = 4; treated: control *n* = 5, LPAR2KO *n* = 4, LPAR3KO *n* = 4, LPAR6KO *n* = 4. Group size: 3D untreated: control *n* = 16, LPAR2KO *n* = 16, LPAR3KO *n* = 16, LPAR6KO *n* = 14; treated: control *n* = 8, LPAR2KO *n* = 9, LPAR3KO *n* = 9, LPAR6KO *n* = 8. (c, d) Protein levels of p-AKT/AKT in ES2 cells. p-AKT/AKT ratio is elevated in LPAR2KO cells both in 2D (*p* < 0.01) and 3D models (*p* < 0.05). In addition, spheroids of LPAR2KO showed decreased p-AKT levels after treatment with 10 *μ*M LPA for 24 hours compared with untreated KO (*p* < 0.05). ^*∗*^*p* < 0.05, ^*∗∗*^*p* < 0.01, and ^*∗*′^*p* < 0.05 compared with untreated cells (Student's *T*-test). Group size: 2D: control *n* = 5, LPAR2KO *n* = 5, LPAR3KO *n* = 5, LPAR6KO *n* = 6; treated: control *n* = 6, LPAR2KO *n* = 5, LPAR3KO *n* = 6, LPAR6KO *n* = 6. Group size: 3D untreated: control *n* = 8, LPAR2KO *n* = 6, LPAR3KO *n* = 10, LPAR6KO *n* = 10; treated: control *n* = 4, LPAR2KO *n* = 4, LPAR3KO *n* = 4, LPAR6KO *n* = 5.

OVCAR3 2D cell lines had elevated levels of p-ERK in LPAR3KO and LPAR6KO cells. In addition, upon LPA treatment, LPAR2KO cells expressed higher levels of p-ERK than treated controls (Figure 2(a)).

On the other hand, highly elevated protein levels of p-ERK were found in LPAR3KO spheroids, yet lower levels were found in LPAR2KO spheroids compared with controls, with no effect seen upon LPA treatment, indicating that both receptors could be involved in the activation of ERK (Figure 2(b)).

### 3.3. LPAR2 Has a Distinct Effect on AKT Activation Levels in ES2 Cells

ES2-LPAR2KO cells had an elevated ratio of p-AKT/AKT in both the 2D and 3D models. LPAR2KO 3D cell treatment with 10 *μ*M LPA resulted in a decrease in AKT activation (Figures 2(c) and 2(d)). This result might indicate that LPAR2 is responsible for AKT activation.

### 3.4. LPAR2, LPAR3, and LPAR6 KO Inhibit OC Invasion and Motility

**Figure 3:**
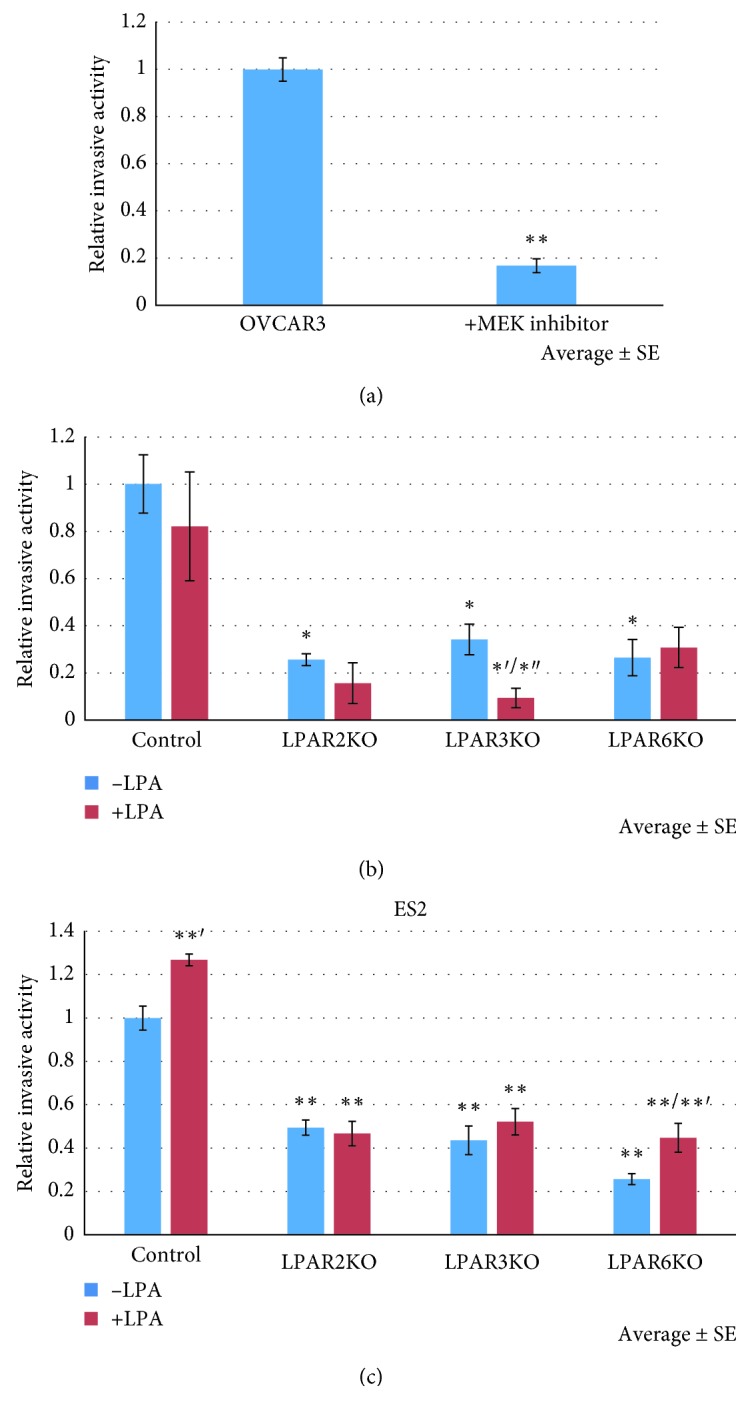
LPAR KO affects invasion. (a) To study the effect of ERK, cells were treated with 0.5 *μ*M of the MEK inhibitor U0126 for 6 hours. The MEK inhibitor U0126 robustly reduced the invasiveness of OVCAR3 cells (*p* < 0.01). Average ± SE; ^*∗∗*^*p* < 0.01 (Student's *t*-test). (b) To study the effect of LPA, cells were treated with 10 *μ*M LPA for 6 hours. All three KO cells expressed a significantly lower invasive activity compared with controls. In LPAR3KO cells, treatment with LPA decreased invasion (group size: *n* = 4 for each group), whereas in LPAR2KO and LPAR6KO cells, there was no statistically different effect (*p* > 0.05). Average ± SE; ^*∗*^*p* < 0.05, ^*∗∗*^*p* < 0.01, and ^*∗*′^*p* < 0.05 compared with treated control and ^*∗*″^*p* < 0.05 compared with untreated KO (Student's *t*-test). (c) Effect of LPARs on invasive activity in ES2 cells. All three KOs decreased invasion in ES2 cells. Cells were treated with 10 *μ*M LPA for 6 hours. LPA had a significant positive effect on invasion in ES2 control and LPAR6KO cells compared with their untreated cells. Average ± SE; ^*∗∗*^*p* < 0.01, ^*∗∗*′^*p* < 0.01 compared with the same untreated group.

To assess the functional consequences of LPAR-KOs on biological processes involved in cancer, the effect of LPA on cellular invasion and migration was tested. To study the effect of ERK on OVCAR3 invasiveness, a MEK 1/2 (ERK1/2 upstream regulating kinases) inhibitor was used. As seen in Figure 3(a), 0.5 *μ*M of the MEK inhibitor U0126 robustly reduced the invasiveness of OC cells, supporting a role for ERK in this process (Figure 3(a)).

**Figure 4:**
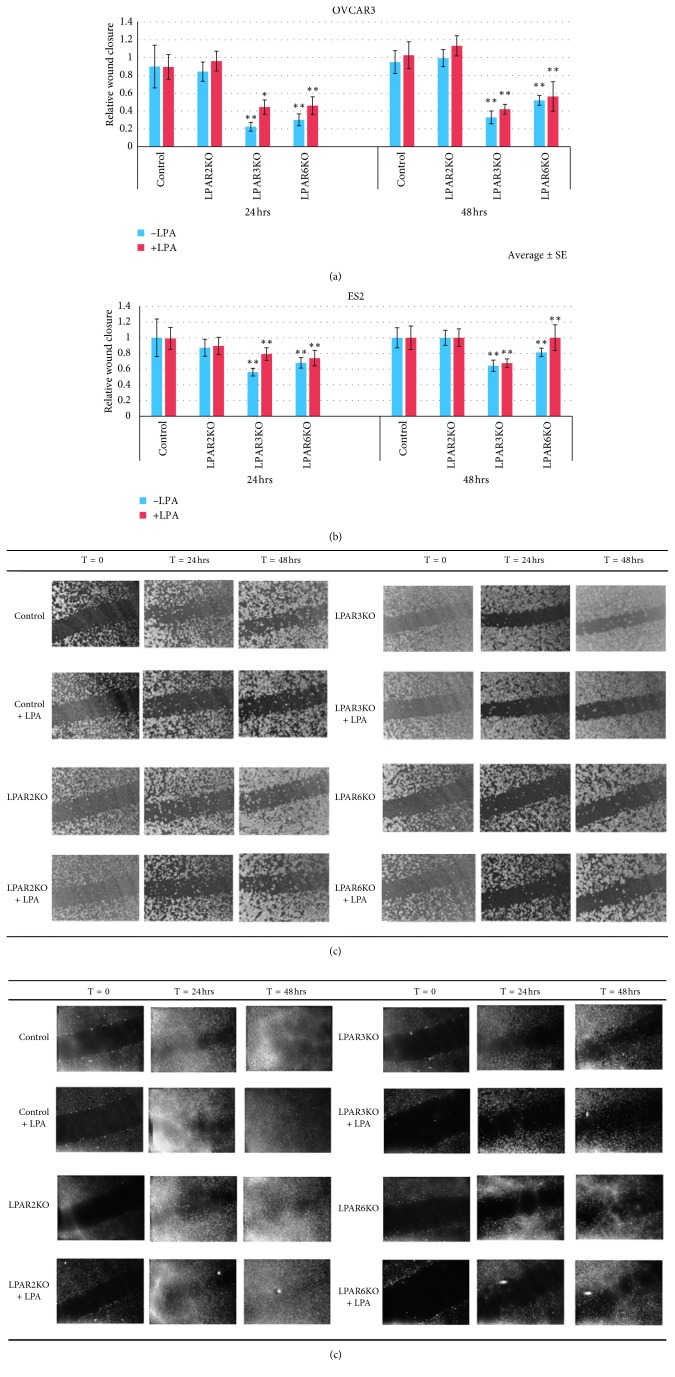
LPAR KO affects motility. Wound healing assay in OVCAR3 and ES2 cultured cells. Cells monolayer cultured in 6-well plates were injured with a sterile 10 *μ*L pipette tip (original wound). Cells were then cultured in DMEM (control) or DMEM containing 10 *μ*M LPA for 48 hours. (a) LPAR3KO and LPAR6KO of OVCAR3 cells show reduced motility compared with control cells in both untreated and LPA-stimulated cells. (b) LPAR3KO and LPAR6KO of ES2 cells show reduced motility compared with control cells both in treated and untreated cells (group size: *n* = 20 for each group). Average ± SE; ^*∗*^*p* < 0.05, ^*∗∗*^*p* < 0.01 (Student's *t*-test). (c, d) The effects of LPAR KO and LPA on cell motility. Images of wound healing assay for OVCAR3 and ES2 cells were taken after 24 h and 48 h, and wound closure was measured.

In OVCAR3 cells, all three LPAR-KOs had significantly reduced invasion compared with controls, yet LPAR3KO cells treated with LPA had reduced invasive ability compared with both control cells and unstimulated LPAR3KO cells (Figure 3(b)). In wound healing assay, LPAR3KO and LPAR6KO cells had reduced motility compared with control cells, both with and without LPA stimulation (Figures 4(a) and 4(c)). The relative wound closure percent was lower in LPAR3KO and LPAR6KO cells at both 24 and 48 hours.

In ES2 cells, there was a significant decrease in invasion in all LPAR KOs with and without treatment with LPA (Figure 3(c)). In addition, LPA induced cell invasiveness in ES2 control cells. In wound healing assay, similar to what we found in OVCAR3 cells, ES2 LPAR3KO and LPAR6KO cells had significantly reduced motility compared with control ES2 cells, both in LPA-treated and untreated cells (Figures 4(b) and 4(d)). The results suggest that LPAR3 and LPAR6, but not LPAR2, contribute to motility of OC cells.

## 4. Discussion

In the present study, we analyzed the signaling pathway of three of the LPARs previously found to be associated with anatomic site and/or survival in HGSC [21]. We combined designing a 3D cell line model and 2D cell cultures with gene editing using the CRISPR/Cas9 method for each of the three studied receptors to determine the main signaling cascade of each one of them and their effect on *in vitro* parameters of tumor aggressiveness.

Our results show that LPAR2 and LPAR3 have opposite effects on the activation of ERK, whereas LPAR6 is responsible for the activation of AKT in OVCAR3 cells. However, knockdown of all three receptors suppressed tumor cell invasion, both in OVCAR3 and ES2 cells.

Using OVCAR3 and the LPAR-KOs, we measured the protein levels of ERK and p-ERK. There was no change in the protein levels of ERK in the KO cells compared with controls. However, the p-ERK/ERK ratio was significantly elevated in LPAR3KO cells cultured both in 2D and 3D form, indicating that LPAR3 pathway is not altered between the two models. LPAR2KO cells expressed low levels of p-ERK/ERK in the 3D form, suggesting that LPAR3 inhibits p-ERK activity downstream, whereas LPAR2 activates it. As for the AKT pathway, LPAR6KO had low p-AKT/AKT ratio when cultured in 2D, but high ratio in the 3D form, suggesting that there might be another factor affecting the phosphorylation of AKT in the 3D model of LPAR6KO cells. However, we found a different pattern in ES2 cells where LPAR2 was apparently related to AKT activation.

Several studies have identified a biological role for LPAR signaling in OC, as well as other cancers. In the study of Ha et al., LPA-induced HIF1*α* activity mediated metabolic reprogramming in OC cells affecting epithelial-to-mesenchymal transition [28]. Yu et al. showed that knockdown of LPAR2 or LPAR3 inhibits the production of IL-6, IL-8, and VEGF in SKOV3 and OVCAR3 cells. Mice with tumors expressing LPAR2 or LPAR3 had reduced survival compared with animals with tumors expressing beta-galactosidase [12]. Recently, Tao et al. analyzed 98 pairs of clinical breast cancer and paratumoral tissues and observed lower expression in the former. Low LPAR6 expression in the tumor was associated with poor prognosis [29].

Wang et al. reported that LPAR2 leads to an increase in LPA-induced uPA activation in SKOV3 cells [30], whereas Jeong et al. demonstrated that LPAR2 is critical for COX-2 expression and inhibits phosphorylation of ERK and EGFR in CAOV-3 cells, affecting cell motility [13]. Seo et al. reported on the role of the ATX-LPA-AKT axis in maintaining OC stem cell-like activity [20]. In the study of Balogh et al., LPAR2 activation led to AKT and ERK phosphorylation in IEC-6 crypt cells [31]. Recently, Park et al. reported that LPA triggers the activation of both ERM and ERK, where LPAR1 and LPAR2 mediate ERM protein activation and cell migration in the OC cell line OVCAR3 [32]. Wang et al. reported that LPA upregulates the expression of the CXCL12-CXCR4 axis, thereby promoting metastasis in SKOV3 and CAOV3 cells [16].

Yu et al. reported that overexpressing LPAR2 and LPAR3 in SKOV3 cells and in nude mice resulted in increased invasiveness and enhanced tumor growth and that overexpression of LPAR2 and LPAR3 further increased their effect on LPA-induced cell migration [12].

We studied the influence of the KO receptors on cell migration using the wound healing assay and observed reduced wound healing in LPAR3KO and LPAR6KO cells compared with controls, a result we found similar in both tested cell lines. Additionally, cell invasion was reduced in all three KO cells. This is in agreement with the previously observed role for these receptors in mediating migration and invasion [12, 19, 33].

In summary, the present study identified a possible signaling pathway for the ATX-LPA axis through LPAR2, LPAR3, and LPAR6 in OC, which affects invasion and motility. ERK and AKT signaling are the major downstream components of this pathway. These results can contribute to understanding the biological mechanisms by which ATX and LPA mediate OC progression. Inhibition of specific LPA receptors may be a novel molecular therapy for this malignancy.

## Figures and Tables

**Table 1:** CRISPR/Cas9 SgRNA.

Gene	SgRNA sequence (5′ ⟶ 3′)

LPAR2	
Forward	CACCGCGGCCCAAGGATGTGGTCG
Reverse	AAACCGACCACATCCTTGGGCCGC

LPAR3	
Forward	CACCGGATCTACGTGTACGTCAAG
Reverse	AAACCTTGACGTACACGTAGATCC

LPAR6	
Forward	CACCGTGTGGTTAACTGTGATCGG
Reverse	AAACCCGATCACAGTTAACCACAC

## Data Availability

The data used to support the findings of this study are available from the corresponding author upon request.
